# A new species and a new record of *Laccaria* (Fungi, Basidiomycota) found in a relict forest of the endangered Fagus grandifolia var. mexicana

**DOI:** 10.3897/mycokeys.27.21326

**Published:** 2017-11-27

**Authors:** Antero Ramos, Victor M. Bandala, Leticia Montoya

**Affiliations:** 1 Red Biodiversidad y Sistemática, Instituto de Ecología, A.C., P.O. Box 63, Xalapa, Veracruz 91000, Mexico

**Keywords:** Ectomycorrhizal fungi, ITS, Neotropical fungi, nLSU, Tricholomatales

## Abstract

Two species of *Laccaria* discovered in relicts of Fagus grandifolia var. mexicana forests in eastern Mexico are described based on the macro- and micromorphological features, and their identity supported by molecular analysis of the internal transcribed spacer (ITS) and large subunit (LSU) of the ribosomal RNA gene. The phylogeny obtained here showed that one of the Mexican species is nested in an exclusive clade which in combination with its striking morphological features, infers that it represents a new species, while the other species is placed as a member in the *Laccaria trichodermophora* clade. This is the first report in Mexico of *Laccaria* with Fagus grandifolia var. mexicana trees, with which the reported species may form ectomycorrhizal association. Descriptions are accompanied with illustrations of macro- and micromorphological characters and a discussion of related taxa are presented.

## Introduction

It has long been recognized that *Laccaria* species are important ectomycorrhizal associates of ectotrophic plants worldwide (Mueller 1992). They are known to form interactions, for example with members of the *Pinaceae*, *Dipterocarpaceae*, *Fagaceae*, *Betulaceae*, *Myrtaceae*, *Tiliaceae* and *Salicaceae* (Kropp and Mueller 1999, Wilson et al. 2013). Some species as *Laccaria laccata* and *L. bicolor* have been considered host-generalists, and inclusive, have been subject of a lot of *in vitro* experimentation worldwide. However, recent studies developed based on molecular systematics showed that under those names, complexes of species are included (Taylor et al. 2006, Jargeat et al. 2010, Vincenot et al. 2012, Sheedy et al. 2013, Popa et al. 2014). A wide ectomycorrhizal host range has also been attributed to *L. amethystina*, but in this case it has some support for its generalist abilities at the population genetics level by Roy et al. (2008), while consideration for cryptic biological species was discarded, at least among the populations sampled in France.

In the monographic work of *Laccaria* by Mueller (1992), 19 species are recognized from North America, and 40 worldwide. New or potential undescribed species from different regions, based on morphological and molecular characteristics of fructifications, or on DNA identifications of environmental samples, have been discovered recently (Wang et al. 2004, Osmundson et al. 2005, Sheedy et al. 2013, Wilson et al. 2013, 2017, Montoya et al. 2015, Luo et al. 2016, Popa et al. 2014, 2016). Nowadays, MycoBank recognizes 112 records in this group of fungi, and additionally, Wilson et al. (2017) inferred 116 phylogenetic species from 30 countries covering the known geographic range of *Laccaria*. During the advances on the systematics of the group, a small number of morphological (macro- and microscopic) features had been found taxonomically informative (McNabb 1972, Mueller 1992), which may be the cause of false interpretations, leading to conceptual misunderstandings. In fact, since early taxonomic studies on the group, the need to revise the species of *Laccaria* commonly treated under names widely cited in the literature was considered as an important task, due to the existence of different, even undescribed species, confused under apparently well-known ones, such as in the groups of *L. laccata* (Scop.) Cooke and *L. proxima* (Boud.) Pat. (Singer 1967, Mueller and Sundberg 1981, Irving et al. 1985). For example, the study by Sheedy et al. (2013) based on DNA multigene sequences, even noted that cryptic phylogenetic species were not nested as sister taxa. Thus, strict species identifications and achieving phylogenetic inferences with stronger resolution in *Laccaria*, will aid in building a robust data set, dealing with each species ectomycorrhizal host range.

In Mexico, the reports of the diversity of the genus *Laccaria* include about 17 species (Aguirre-Acosta and Pérez-Silva 1978, Bandala et al. 1988, Montoya et al. 1987, 2015, Cifuentes et al. 1990, Pérez-Silva et al. 2006, Garibay-Orijel et al. 2009). The edibility and use of some species as food has been documented (e.g. Montoya et al. 1987, Montoya-Esquivel et al. 2002, 2003, Lampman 2007, Pérez-Moreno et al. 2008) and ectomycorrhizae formed under *in vitro* culture conditions, isolated from native specimens have also been achieved (Santiago-Martínez et al. 2003, Carrasco-Hernández et al. 2010, Galindo-Flores et al. 2015). Molecular studies on most of those records are needed not only to support their identifications but for being included in phylogenetic studies. *Laccaria roseoalbescens* T. J. Baroni, Montoya and Bandala, described as new (Montoya et al. 2015) from the mesophytic forest in Veracruz, was recognized under morphological features and confirmed through phylogenetic DNA sequence analyses and recently incorporated by Luo et al. (2016) in their molecular phylogeny to confirm the distinction of the new *L. rubroalba* X. Luo, L. Ye, Mortimer & K.D. Hyde from China.

We have under research the fungal community associated to the two southernmost relicts of mesophytic forests dominated by Fagus grandifolia var. mexicana in the American Continent. This tree species is currently in danger of extinction and in the Red list of Mexican cloud forest trees, inhabiting a narrow range of nearly 145 hm^2^ in Mexico (Rodríguez-Ramírez et al. 2013, Montoya et al. 2017). Taking into account its current status, we consider important to document the associated fungal species with particular focus to the ectomycorrhizal forming species. During our study, we found two species of *Laccaria* which after their morpho- and molecular analyses we concluded that with strong support can be recognized, one as *L. trichodermophora* G.M. Mueller and the other, as a distinct undescribed species close to *L. angustilamella* Zhu, L., Yang & L. Wang from China. As both are part of the unknown potential mycobionts of this endangered ectotrophic tree species, we were motivated to document them.

## Materials and methods

### Sampling and morphological study of basidiomes

Random visits were conducted during August–September 2005 and 2007, in two stands of Fagus grandifolia var. mexicana from Veracruz, Mexico, one in Acatlán Volcano, Acatlán (19°40'43.9"N; 96°51'9.8"W, 1840 m) and the other in Mesa de la Yerba, Acajete (19°33'37.2"N; 97°01'9.8"W, 1900 m). Basidiomes of *Laccaria* growing close to *Fagus* were gathered. Macromorphological characters and color were recorded, alphanumeric color codes in descriptions refer to Kornerup and Wanscher (1967). Measurements and colors of micromorphological structures were recorded in 3% KOH. Basidiospores were studied in Melzer’s reagent. Methods to determine spore ranges are those used by Montoya and Bandala (2003), with 45–50 spores measured per collection (length and width of the sporoid excluding the ornamentation) and given as a range with the symbol *X̅* representing mean values. *Q̅* represents the basidiospore length/width ratio and is given as range of mean values. Line drawings were made with a drawing tube. The examined specimens studied are deposited in XAL herbarium (acronym from B. Thiers, continuously updated; Index Herbariorum: http://sweetgum.nybg.org/ih/). The SEM images were obtained after critical point drying of pieces of lamellae previously rehydrated in ammonia, fixed in glutaraldehyde and dehydrated in an ethanol series (Bandala and Montoya 2000).

### DNA extraction, PCR amplification, and sequencing

Genomic DNA of the Mexican specimen was extracted according to Montoya et al. (2014). PCR was performed to amplify the ITS (Internal Transcribed Spacer) and LSU (Large Subunit) regions of the nuclear rDNA, using primers ITS1F, ITS5/ITS4, LR0R/LR21, LR7 (Vilgalys and Hester 1990, White et al. 1990, Gardes and Bruns 1993). PCR conditions, as well as procedures for the purification of amplified PCR products, cycle sequencing reactions and their purification were done according to Montoya et al. (2014). Once sequences were assembled and edited, they were deposited at GenBank database (Benson et al. 2017) (Table 1).

**Table 1.** *Laccaria* taxa included in this study: samples, location and GenBank accession number for sequences.

Taxon	Voucher	Location	GenBank	
			ITS	28S
*Cortinarius violaceus*	MTS 4854 (WTU)	USA: Washington	DQ486695	DQ457662
*L. alba*	AWW438	China: Yunnan-Shangrila	JX504094	JX504178
*L. alba*	F1120750	China	JX504126	JX504206
*L. alba*	F1121461	China	JX504129	JX504209
*L. alba*	GMM6131	China: Chang Bai Shan	JX504131	JX504210
*L. amethystea*	FP-98556	Germany: Vorpommern	DQ499640	–
*L. amethystea*	TUB 011464	Germany	AF539737	–
*L. amethysteo-occidentalis*	AWW556	USA: California, Nevada Co.	JX504107	JX504191
*L. amethysteo-occidentalis*	AWW590	USA: Oregon, Benton Co.	JX504112	JX504195
*L. amethystina*	ALB183	China: Tibet	JX504092	JX504176
*L. amethystina*	F1123822	USA: Wisconsin	KU685760	KU685911
*L. amethystina*	GMM7041	Russia: Caucasus	KU685654	KU685797
*L. amethystina*	GMM7621	France: Forest comaniale de Ste. Croix	JX504150	JX504224
*L. amethystina*	LaAM-08-1	–	JGI Genome	JGI Genome
*L. angustilamella*	BAP226	China: Yunnan	JX504118	JX504201
*L. angustilamella*	HKAS58714	China: Yunnan, Yongping	JX504168	JX504244
*L. aurantia*	KUN-F 78557-Type	China: Yunnan	JQ670895	–
*L. aurantia*	MB-FB-101109	China: Yunnan	JQ681209	–
*L. bicolor*	AWW539	USA: Illinois	KM067817	KU685763
*L. bicolor*	AWW537	USA: Illinois, Johnson Co.	JX504105	JX504189
*L. major*	GMM6012	Costa Rica	KU685758	KU685909
*L. major*	GMM6019	Costa Rica	KU685757	KU685908
*L. nobilis*	F1091206	USA: Michigan	KU685636	KU685779
*L. ochropurpurea*	JMP0038	USA: Wisconsin	EU819479	–
*L. ochropurpurea*	KH_LA06_016	USA: Louisiana	KU685721	–
*L. ochropurpurea*	PRL3777	USA: Illinois	KU685732	JX504246
*L. ochropurpurea*	PRL4777	USA: Illinois	KU685733	KU685883
*L. proxima*	F1133825	USA: Mississippi	KU685642	KU685786
*L. roseoalbescens*	LM5042	Mexico: Veracruz	KJ874327	KJ874330
*L. roseoalbescens*	LM5099-Type	Mexico: Veracruz	KJ874328	KJ874331
*L. salmonicolor*	GMM7596-Type	China: Tibet	JX504143	JX504218
*L. salmonicolor*	GMM7602	China: Tibet	JX504145	JX504220
*L.* sp.	A0561	Japan: Sapporo	JX504082	–
*L.* sp.	A0573	Japan: Narusawa	KU685617	–
*L.* sp.	GMM6800	Guatemala	KU685756	KU685907
*L. squarrosa* ^a^	DM121	Mexico: Veracruz	MF669960	MF669967
*L. squarrosa* ^a^	DM63-Type	Mexico: Veracruz	MF669958	MF669965
*L. squarrosa* ^a^	DM93	Mexico: Veracruz	MF669959	MF669966
*L. trichodermophora*	TENN42523-Type	USA: Texas	DQ149868	–
*L. trichodermophora*	F1111951	Costa Rica	KU685640	KU685784
*L. trichodermophora*	GMM7733	USA: Texas, Tyler Co.	JX504157	JX504230
*L. trichodermophora*	KH_LA06_013	USA: Louisiana	KM067881	KU685872
*L. trichodermophora*	GMM7735	USA: Texas	KM067872	–
*L. trichodermophora*	KH-LA06-012	USA: Louisiana	KM067880	–
*L. trichodermophora*	GMM7734	USA: Texas	KM067871	–
*L. trichodermophora*	KH-LA06-007	USA: Louisiana	KM067874	–
*L. trichodermophora*	KH-LA06-008	USA: Louisiana	KM067875	–
*L. trichodermophora*	tri1125225	USA: Rocky Mountains	DQ149855	–
*L. trichodermophora*	KH-LA06-010	USA: Louisiana	KM067878	–
*L. trichodermophora*	KH-LA06-011	USA: Louisiana	KM067879	–
*L. trichodermophora*	KH-LA06-009	USA: Louisiana	KM067876	–
*L. trichodermophora*	KH-LA06-009B	USA: Louisiana	KM067877	–
*L. trichodermophora*	KH-LA06-004	USA: Louisiana	KM067873	–
*L. trichodermophora*	HC-PNNT-112	Mexico: Mexico State	KT875031	–
*L. trichodermophora*	GO-2009-266	Mexico: Mexico State	KC152147	–
*L. trichodermophora*	HC-PNNT-157	Mexico: Mexico State	KT875032	–
*L. trichodermophora*	GO-2009-305	Mexico: Distrito Federal	KC152149	–
*L. trichodermophora*	GO-2010-124	Mexico: Veracruz	KC152144	–
*L. trichodermophora*	EF36	Mexico	KT354980	–
*L. trichodermophora*	CB08167	Mexico: Mexico State	KT875029	–
*L. trichodermophora*	GO-2009-228	Mexico: Mexico State	KC152146	–
*L. trichodermophora*	GO-2010-126	Mexico: Veracruz	KC152145	–
*L. trichodermophora*	GO-2010-082	Mexico: Tlaxcala	KC152152	–
*L. trichodermophora*	GO-2009-225	Mexico: Mexico State	KC152143	–
*L. trichodermophora*	GO-2009-484	Mexico: Tlaxcala	KC152151	–
*L. trichodermophora*	HC-PNNT-192	Mexico: Mexico State	KT875033	–
*L. trichodermophora*	GO-2009-210	Mexico: Mexico State	KC152148	–
*L. trichodermophora*	HC-PNNT-132	Mexico: Mexico State	KT875030	–
*L. trichodermophora*	GO-2009-314	Mexico: Jalisco	KC152150	–
*L. trichodermophora*	HC-PNNT-099	Mexico: Mexico State	KT875034	–
*L. trichodermophora*	GMM7714	USA: Texas	KM067867	–
*L. trichodermophora*	GMM7712	USA: Texas	KM067866	–
*L. trichodermophora*	GMM7716	USA: Texas	KM067869	–
*L. trichodermophora*	HMJAU26938	–	KP128033	–
*L. trichodermophora*	GMM7703	USA: Texas	KM067865	–
*L. trichodermophora*	GMM7697	USA: Texas	KM067863	–
*L. trichodermophora*	GMM7698	USA: Texas	KM067864	–
*L. trichodermophora*^a^	Montoya 4393	Mexico: Veracruz	MF669961	MF669968
*L. trichodermophora*^a^	Montoya 4394	Mexico: Veracruz	MF669962	MF669969
*L. trichodermophora*^a^	AR24	Mexico: Veracruz	MF669964	MF669970
*L. trichodermophora*^a^	Bandala 4282	Mexico: Veracruz	MF669963	–
*Psathyrella rhodospora*	MP133 MN	–	DQ267129	AY645058

^a^samples and sequences obtained here.

### Phylogenetic methods

The phylogenetic analysis was performed with the sequences obtained in this study, as well as some retrieved from GenBank (http://www.ncbi.nlm.nih.gov/) derived from the Blast analysis (only those that best match), and complemented with related sequences used by Osmundson et al. (2005), Montoya et al. (2015) and Wilson et al. (2017) (Table 1). For this purpose, we constructed a dataset (ITS+LSU) using PhyDE v.0.9971 (Müller et al. 2010), also with MEGA 6.06 (Tamura et al. 2013) calculated the best evolutionary model and constructed the phylogenetic tree under the method of Maximum Likelihood (ML) with 500 bootstrap replications, and finally with MrBayes v 3.2.6 (Ronquist et al. 2012) constructed the phylogenetic tree (as Montoya et al. 2014) under the method of Bayesian Inference (BI). The phylogenies from ML and BI analyses were displayed using Mega 6.06 and FigTree v1.4.3 (Rambaut 2016) respectively.

## Results

**Figure 1.**
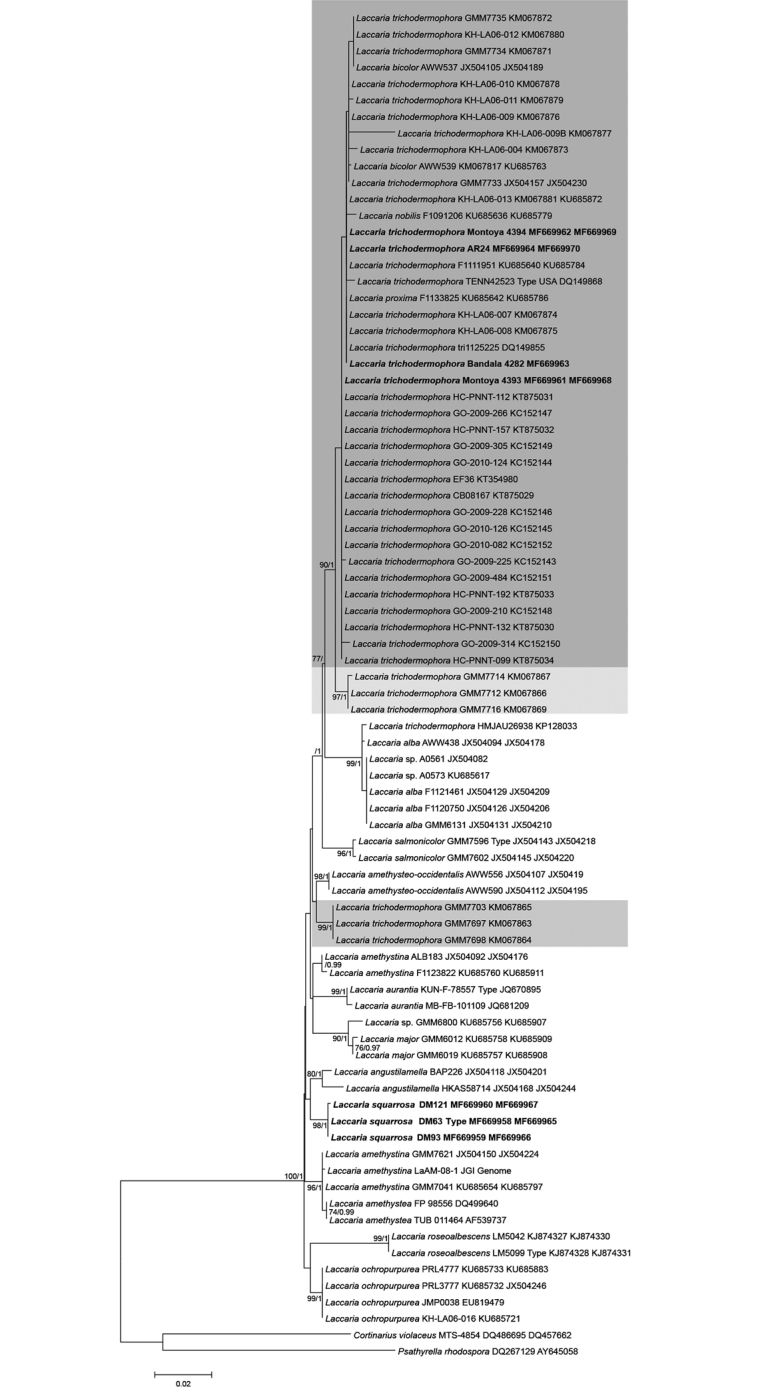
Phylogenetic relationships within *Laccaria* species inferred from the combined ITS and LSU sequence data by maximum likelihood method. Tree with the highest log likelihood (–4163.7219), the percentage of trees in which the associated taxa clustered together (only values ≥ 70% are considered) is shown next to the branches, followed by the posterior probabilities (only values ≥ 0.90 are indicated) obtained after Bayesian inference. The tree is drawn to scale, with branch lengths measured in the number of substitutions per site.

A total of 13 new ITS and 28S sequences for *Laccaria* were generated in this study (Table 1 and alignment in TreeBASE S21413). They were obtained from *Laccaria* samples proceeding from the two stands of Fagus grandifolia var. mexicana in the subtropical cloud forest in central Veracruz (sample AR24 comes from a conifers forest in Veracruz) (Table 1). Only bootstrap values of ≥70% and posterior probabilities (ML/PP) of ≥0.90 were considered and indicated on the tree branches. The phylogeny displayed (Fig. 1) inferred the Mexican samples clustered in two distinct clades. A group clearly related to *Laccaria trichodermophora* and another, in a separate clade, representing an undescribed species.

## Taxonomy

### Laccaria squarrosa

Taxon classificationFungiAgaricalesHydnangiaceae

Bandala, Montoya & Ramos sp. nov.

823034

Figs 2 , 3 , 4 , 5

#### Holotype.

MEXICO, Veracruz State, Mpio. Acatlán, Volcán de Acatlán, Aug 14 2007, DM 63 (XAL). Terrestrial under Fagus grandifolia var. mexicana.

#### Diagnosis.

*D*iffers from other species by having medium sized basidiomes, with pinkish to pale brownish-orange colors, smooth to finely squarrose surfaces, especially on the stipe, basal mycelium with whitish to pale brownish with pinkish tinges, and globose, echinulate basidiospores, 7–10 (-11.5) × 7–10.5 µm, with the echinulae 0.5–1.4 in length, 0.45–0.9 µm in width at base, subcylindrical to contorted cystidia and pileipellis arranged in a cutis with mounds of intermixed and irregularly projected hyphae.

**Figure 2.**
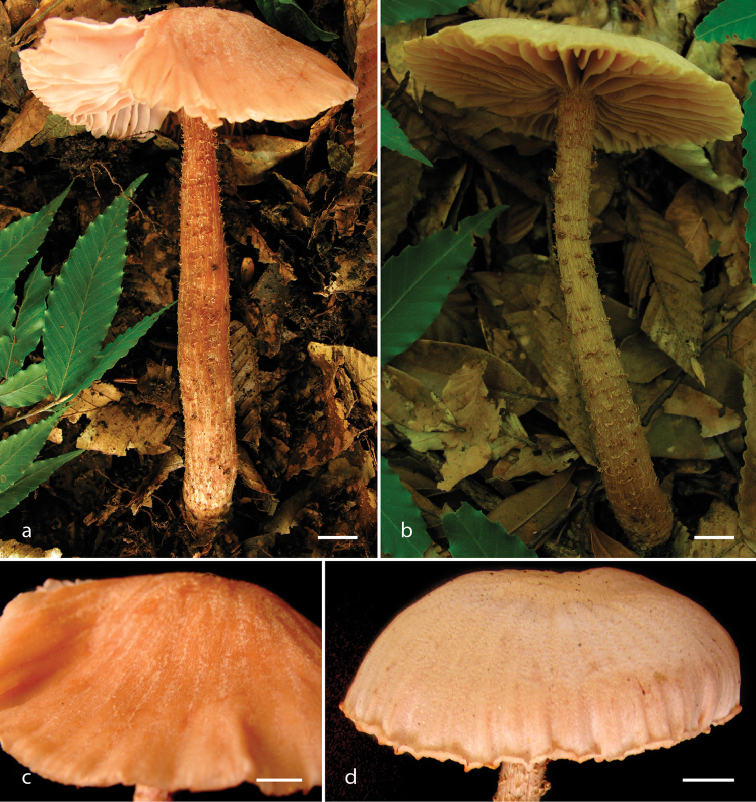
*Laccaria squarrosa*, basidiomes. **a, b** habit **c, d** pileus surface details **a, c** DM 121 **b** DM 63 (holotype) **d** DM 93. Scale bars: 10 mm.

**Figure 3.**
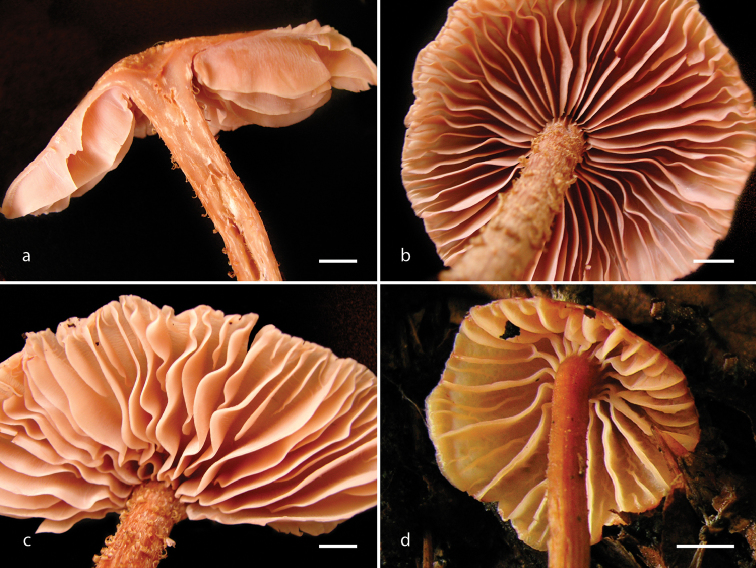
*Laccaria squarrosa*, lamellae attachment and habit. **a, c** DM 121 **b** DM 63 (holotype) **d** DM 93. Scale bars: 10 mm.

**Figure 4.**
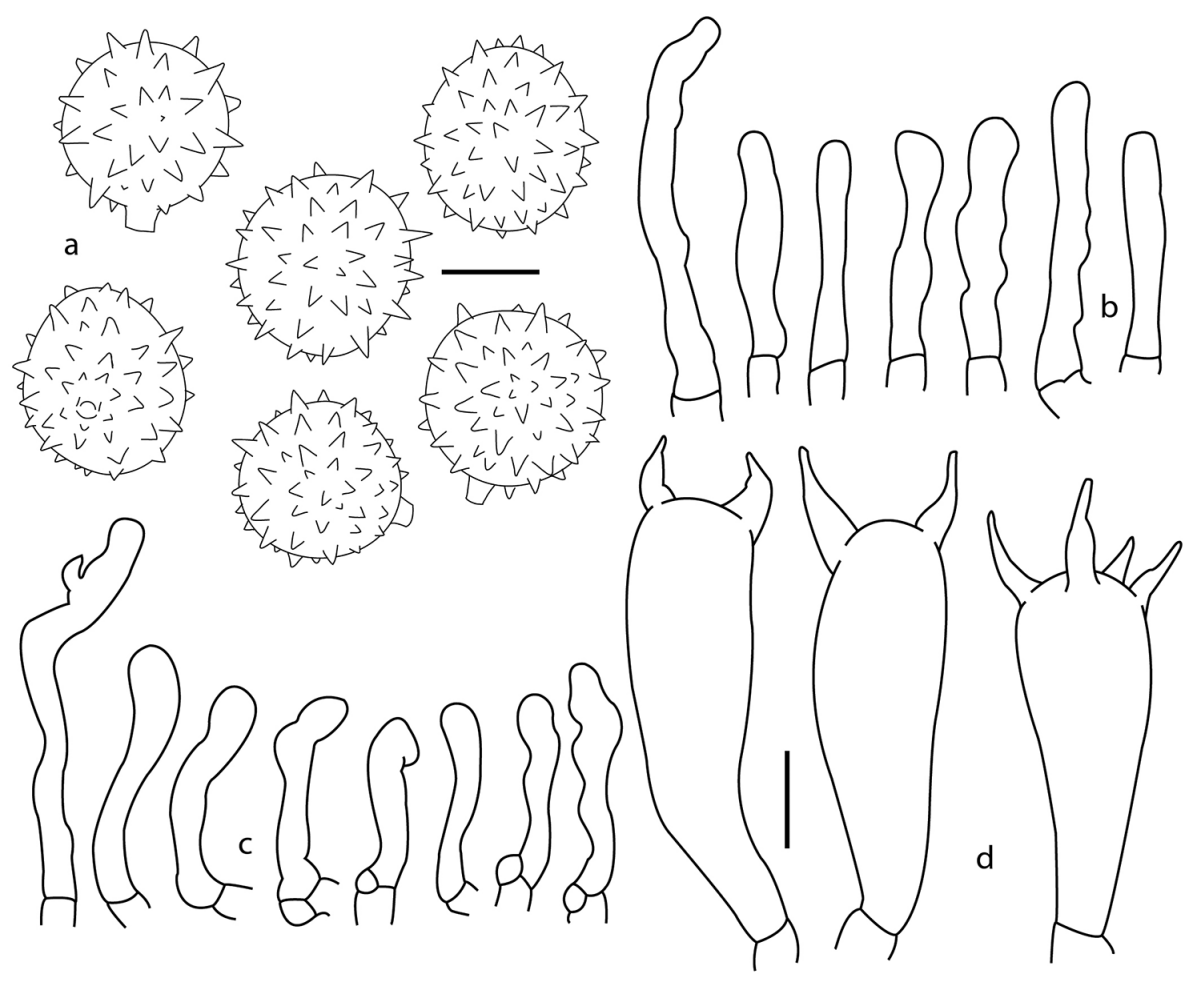
*Laccaria squarrosa*, **a** basidiospores **b** pleurocystidia **c** cheilocystidia **d** basidia **a, c** DM 121 **b** DM 63 (holotype) **d** DM 93. Scale bars: 5 µm (**a**); 10 µm (**b–d**).

**Figure 5.**
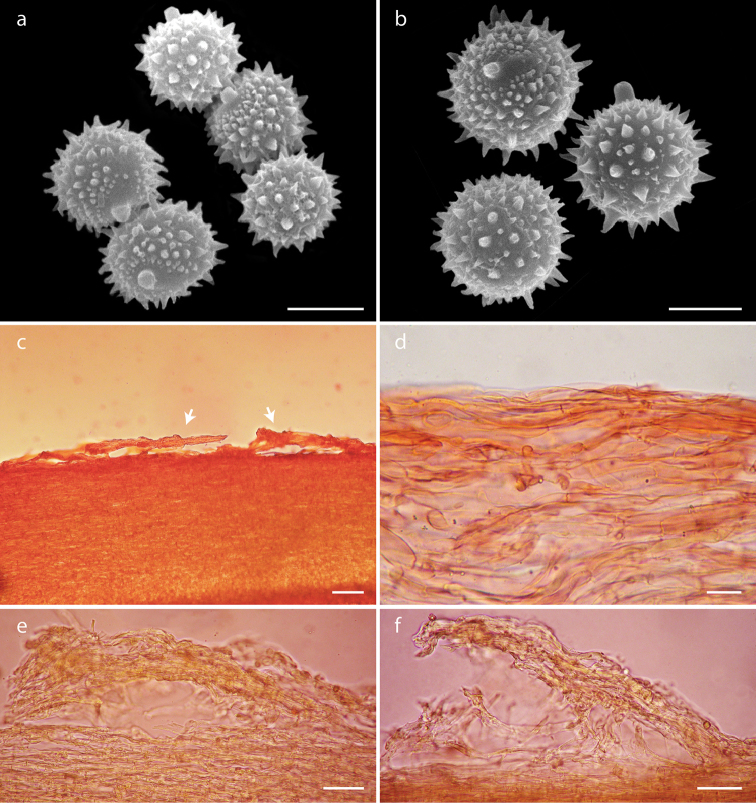
*Laccaria squarrosa*, **a–b** basidiospores under SEM **c–f** details of the pileipellis **c–d** cutis (arrow indicating scales) **e–f** details of the pileipellis scales **c–f** DM 63 (holotype). Scale bars: 5 µm (**a**); 2 µm (**b**); 100 µm (**c**); 20 µm (**d**); 50 µm (**e–f**).

#### Gene sequences ex-holotype.

MF669958 (ITS), MF669965 (LSU).

#### Etymology.

referring to the characteristic squarrose surfaces of basidiomata.

*Pileus* 10–82 mm diam convex to plane-convex, at times slightly depressed at center, surface squamulose to squarrose with age, pinkish (6B3–2) with pale yellowish tinges towards the center or brownish-orange (5B6–5) when young; margin recurved, striate, edge thin. *Lamellae* 1–8 mm in length, adnate to subdecurrent, at times slightly undulate, subdistant or distant, pinkish to pale pinkish (6B4), 1–2 lamellulae per lamellae of different sizes. *Stipe* 50–155 × 5–9 mm, cylindrical, widened towards the base, squamulose to squarrose overall, more densely scaly towards the apex and when old, squamules brown, pinkish to ochraceous or ochraceous-orange. *Basal mycelium* pale whitish to brownish (6D6), with pinkish tinges in some areas. KOH negative overall surfaces.

*Basidiospores* 7–10 (-11.5) × 7–10.5 µm, *X̅*=7.8–10.7 × 7.7–9.48 µm, *Q̅*=1.01–1.12, globose, pale brownish, thin walled, hyaline, inamyloid, echinulate; under SEM the echinulae appear acute, 0.5–1.4 in length, 0.45–0.9 µm in width at base, shorter towards the hylar appendix area, this latter structure (also called the apiculus) consisting of a tube with rounded ending. *Basidia* 35–66 × 10–15 µm, clavate to narrowly clavate, thin walled, mostly tetrasporic, at times tri- or bisporic, sterigmata 10 µm length, some with refringent contents, clamped, hyaline. *Pleurocystidia* 20–38 × 3–6 µm, subcylindrical, contorted, sinuous, hyaline, thin walled. *Cheilocystidia* 14–40 × 2–5 µm, subcylindrical, rarely narrowly utriform, contorted, sinuous, hyaline, thin walled. *Pileipellis* a regular compact cutis, hyphae periclinally oriented, also with projected mounds of intermixed hyphae, which form the pileus scales irregularly projected; hyphae cylindrical, some widened 4–10 µm diam, some septate, hyaline, inamyloid, yellowish in mass and somewhat refringent in some parts, thin walled, clamped. *Context hyphae* cylindrical, faintly yellowish in mass, 4–9 (-14) µm diam, thin walled, up to 1 µm diam, hyaline, inamiloyd, septate. *Hymenophoral trama* regular, compact, composed by cylindrical hyphae, pale yellowish in mass, 3–8 µm diam, septate, hyaline and inamyloid, thin walled. *Clamps* present.

#### Habitat.

Terrestrial, solitary, under Fagus grandifolia var. mexicana.

#### Additional studied material.

MEXICO, Veracruz, Mpio. Acatlán, Volcán de Acatlán, Sep 18 2007, DM 121. Mpio. Acajete, Mesa de la Yerba, Aug 28 2007, DM 93 (all at XAL).

## Discussion

In the phylogeny presented here that is based on sequences used in the worldwide survey of *Laccaria* by Wilson et al. (2017) and complemented with some from GenBank (Fig. 1) and sequences of *L. squarrosa*, described here, this new taxon is clearly shown to be phylogenetically isolated from other *Laccaria* species. *Laccaria squarrosa* is distinct by possessing typical medium sized basidiomes with scaly surfaces, more obvious especially on the stipe and by having the basal stipe mycelium whitish to pale brownish with pinkish tinges. Microscopically it differs by globose, echinulate basidiospores, cylindrical cystidia and pileipellis arranged in a cutis with mounds of intermixed and irregularly projected hyphae. In Fig. 1, *L. squarrosa* is shown to be phylogenetically close to *L. angustilamella* Zhu L., Yang & L. Wang from China. This later species is characterized, however, by having a marasmioid to mycenoid habit, with a short basidiome size (pileus 20–30 mm diam), narrow (2 mm length) and subdistant lamellae, non-scaly stipe, with more ellipsoid basidiospores (Q up to 1.18) and larger echinulae (2.0–) 2.5–5.0 µm long and up to 2.5 µm wide at base (Wang et al. 2004).

Color features of the basidiomes and whitish mycelia relate *Laccaria squarrosa* to metasection *Laccaria* (Mueller 1992), where it superficially resembles *L. proxima* (Boudier) Patouillard. This later species, however, can be distinguished based on the longitudinally striate stipe, with a fibrillose surface only, ellipsoid basidiospores [9–11.5 × 6.7–8 (-8.8) µm, Q = 1.25–1.35 (-1.4)], having shorter echinulae (0.5–1 µm length), pleurocystidia absence and larger cheilocystidia [19–66.5(-92) × 2–8.5(-16.5) µm] (Mueller 1992). Among the species in the genus, *Laccaria nobilis* A.H. Smith, *L. amethysteo-occidentalis* G.M. Muell., *L. trichodermophora* and *L. ochropurpurea* (Berk.) Peck also produce fibrillose to somewhat scaly pileus surfaces. *Laccaria ochropurpurea* even can have recurved scales on the stipe surface. However, all those taxa clearly differ from *Laccaria squarrosa* by basidiomes and mycelia with violaceous colors, besides other macro and microscopical features (Mueller 1992).

**Figure 6.**
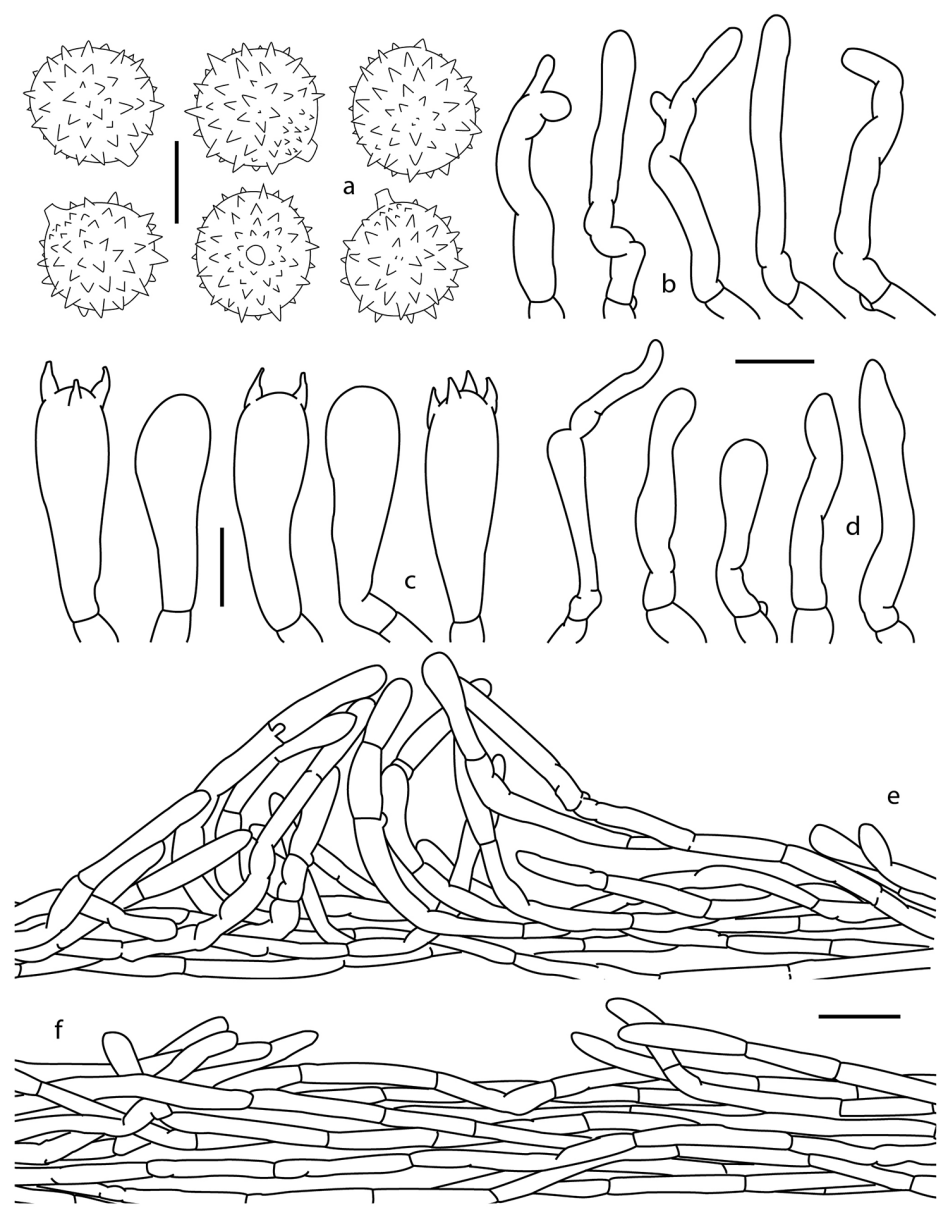
*Laccaria trichodermophora*, **a** basidiospores **b** pleurocystidia **c** basidia **d** pileipellis **e** cheilocystidia **a–e** Montoya 4393. Scale bars: 5 µm (**a**); 10 µm (**b–c, e**); 25 µm (**d**).

**Figure 7.**
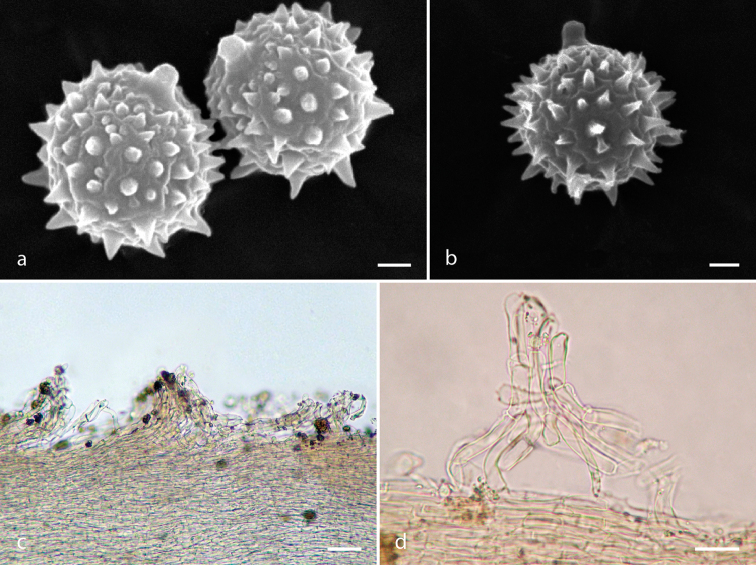
*Laccaria trichodermophora*, **a–b** basidiospores under SEM **c–d** pileipellis **a, c–d** Montoya 4393 **b** Bandala 4282. Scale bars: 1 µm (**a–b**); 50 µm (**c**); 25 µm (**d**).

*Laccaria trichodermophora* G.M. Mueller (Figs 6–7) was previously reported from Mexico (as *L. farinacea* sensu Singer) by Montoya et al. (1987) from conifer forests of Cofre de Perote National Park areas. The collections from Fagus grandifolia var. mexicana forest here studied, were collected in the locality of Mesa de la Yerba (Veracruz), on Aug 04 2005, Montoya 4393, Montoya 4394; Aug 28 2007, Bandala 4282 (XAL). Excepting by narrower hyphae disposed in the pileipellis mounds and the basidiospores including broadly ellipsoid to ellipsoid shapes, exhibit a similar morphological variation as those described by Mueller (1984, 1992) and other collections reported before from Mexico. A summary of the main morphological features that characterize the studied materials are: *pileus* 15–55 mm diam, fibrillose to fibrillose-minutely scaly, brownish-orange (6C6–C7), light brown or pale pinkish-brown or pale brownish towards the margin (6D6, 7C5–C4), hygrophanous. *Lamellae* 2‒6 mm in length, adnate to sinuate, close to subdistant, pinkish or incarnate (6A2‒B3). *Stipe* 20–75 × 2–8 mm, cylindrical, attenuated towards the apex, striate, fibrillose and fistulous. hygrophanous, concolorous to pileus but more pinkish-violaceous (13CD2) towards the base. *Basal mycelium* violaceous becoming white. *Basidiospores* 6–9 (-10) × 6–8.5 (-10) µm, *X̅*= 6.9–8.18 × 6.84–7.9 µm, *Q̅*= 1.00–1.05, globose, hyaline, echinulate, under SEM the echinulae appear 0.8–1.13 × 0.6–0.8 µm. *Basidia* 27–65 (-80) × 7–13 µm, clavate, tetra or at times tri-sporic, hyaline, thin walled, clamped. *Cheilocystidia* 12–49 × 2–6 µm cylindrical to narrowly clavate, at times somewhat utriform, hyaline, thin walled, frequently clamped. *Pileipellis* composed of periclinally oriented hypahe 3–10 µm diam, in a more or less cutis arrangement but with frequent mounds of intermixed or erect hyphae, with terminal elements 25–125 × 10–13 µm, cylindrical to clavate other somewhat utriform 20–65 × 5–17 µm. *Clamps present*.

In the phylogeny obtained (Fig. 1), the sampled sequences of this species appear in three clades. One of them, with collections from North America, included the type specimen (DQ149868) and 21 specimens from Mexico. Our collections clustered in this later lineage interestingly with one sample from Costa Rica too. The other two clades are composed of specimens from Texas, one of them sister to the type clade, and the third clearly separated, probably representing an undescribed species. A specimen (KP128033) labeled as *L. trichodermophora* in the GenBank, clustered in *L. alba* group from Asia in our analysis. This sample lacks geographic information and could well be a misidentified collection.

There are no previous reports of *Laccaria trichodermophora* being associated with *Fagus grandifolia var. mexicana*. This report serves as the first documentation of this association. According to the reports of *L. trichodermophora*, it shows a wide ecological range. Mueller (1992) observed that all collections of this *Laccaria* species from the southeastern United States appeared to be associated with *Pinus*. He also collected it, in Costa Rica, beneath Neotropical species of *Quercus*. In central Mexico, in the states of Tlaxcala and Michoacán, it has been recorded associated to mixed *Pinus*-*Alnus* and *Pinus*-*Abies* forests (Montoya et al. 1987, Montoya-Esquivel et al. 2004). In the eastern part of Mexico, in Veracruz, it has been found (as *L. farinacea* sensu Singer) in monodominant *Pinus* and mixed *Pinus*-*Abies* forests (Montoya et al. 1987). In this later country, it is interesting to note that, basidiomes of this species, specially from conifers, are sold in local markets as edible fungi (Montoya et al. 1987, Montoya-Esquivel et al. 2004). Based on the available ecological information of the samples in the phylogenetic tree (Fig. 1), a wide host range for *L. trichodermophora* type specimen clade can be inferred. Among the potential hosts, it can be recognized as occurring with *Fagus grandifolia*, *Pinus elliottii*, *P. palustris* and *Quercus* sp. in Texas, as well as *P. patula*, other species of Pinaceae and *Quercus* spp. in both US and in Mexico, and the endangered F. grandifolia var. mexicana as confirmed here. *Abies religiosa* represents another host also, as proved by data from two sequences (MF669964 and MF669970) (Table 1, Fig. 1) obtained here, from the sample AR24, from an *A. religiosa* forest at Cofre de Perote National Park in Veracruz, Mexico.

## Supplementary Material

XML Treatment for Laccaria squarrosa
